# The Intersection of Neighborhood Environment and Adverse Childhood Experiences: Methods for Creation of a Neighborhood ACEs Index

**DOI:** 10.3390/ijerph19137819

**Published:** 2022-06-25

**Authors:** Krista Schroeder, Levent Dumenci, David B. Sarwer, Jennie G. Noll, Kevin A. Henry, Shakira F. Suglia, Christine M. Forke, David C. Wheeler

**Affiliations:** 1 Department of Nursing, Temple University College of Public Health, Philadelphia, PA 19122, USA; 2 Department of Epidemiology and Biostatistics, Temple University College of Public Health, Philadelphia, PA 19122, USA; ldumenci@temple.edu; 3 Department of Social and Behavioral Sciences, Center for Obesity Research and Education, Temple University College of Public Health, Philadelphia, PA 19122, USA; dsarwer@temple.edu; 4 Department of Human Development and Family Studies, Penn State College of Health and Human Development, University Park, PA 16802, USA; jgn3@psu.edu; 5 Department of Geography and Urban Studies, Temple University College of Liberal Arts, Philadelphia, PA 19122, USA; kevinahenry@temple.edu; 6 Department of Epidemiology, Emory University Rollins School of Public Health, Atlanta, GA 30322, USA; shakira.suglia@emory.edu; 7 Master of Public Health Program, Perelman School of Medicine, University of Pennsylvania, Center for Violence Prevention, Children’s Hospital of Philadelphia, Philadelphia, PA 19104, USA; forkeyoungc@chop.edu; 8 Department of Biostatistics, Virginia Commonwealth University School of Medicine, Richmond, VA 23298, USA; david.wheeler@vcuhealth.org

**Keywords:** adverse childhood experiences, geospatial, index, methods, neighborhood, obesity, spatial, trauma, neighborhood ACEs index

## Abstract

This study evaluated methods for creating a neighborhood adverse childhood experiences (ACEs) index, a composite measure that captures the association between neighborhood environment characteristics (e.g., crime, healthcare access) and individual-level ACEs exposure, for a particular population. A neighborhood ACEs index can help understand and address neighborhood-level influences on health among individuals affected by ACEs. Methods entailed cross-sectional secondary analysis connecting individual-level ACEs data from the Philadelphia ACE Survey (*n* = 1677) with 25 spatial datasets capturing neighborhood characteristics. Four methods were tested for index creation (three methods of principal components analysis, Bayesian index regression). Resulting indexes were compared using Akaike Information Criteria for accuracy in explaining ACEs exposure. Exploratory linear regression analyses were conducted to examine associations between ACEs, the neighborhood ACEs index, and a health outcome—in this case body mass index (BMI). Results demonstrated that Bayesian index regression was the best method for index creation. The neighborhood ACEs index was associated with higher BMI, both independently and after controlling for ACEs exposure. The neighborhood ACEs index attenuated the association between BMI and ACEs. Future research can employ a neighborhood ACEs index to inform upstream, place-based interventions and policies to promote health among individuals affected by ACEs.

## 1. Introduction

Adverse childhood experiences (ACEs) are traumatic events that occur before the age of 18 years. Experiences commonly considered ACEs include physical, sexual, or emotional abuse, physical or emotional neglect, witnessing domestic or community violence, and a household member’s substance use, incarceration, or mental illness [1,2,3]. Estimates of ACEs prevalence vary across populations, though most suggest that at least half to two-thirds of individuals have experienced one or more ACEs, with members of groups affected by health inequities bearing a disproportionate ACEs burden [2,4,5,6,7,8,9]. ACEs are associated with health-hindering behaviors and worse mental and physical health [5,6,8], including smoking, alcohol use, anxiety, depression, cancer, and cardiovascular disease. ACEs’ effects vary based upon ACE type, severity, chronicity, as well as how the ACEs are experienced by the affected individual. Further, ACEs can be mitigated by supportive resources, protective factors, and resilience, leading to individual variation in harmful effects of ACEs [5,6,8,10]. However, in general, ACEs-associated risks increase with number of ACEs experienced. In particular, experiencing 4+ ACEs is a threshold associated with markedly worse health outcomes [5,11].

Obesity, defined as a body mass index (BMI) ≥ 30 kg/m^2^, is one of the health outcomes associated with ACEs. The association of ACEs with BMI in adulthood is well characterized [5,12,13,14,15,16,17,18,19,20,21,22,23,24]. Research examining association of ACEs with elevated BMI and obesity during childhood is sparser and less consistent, though recent work suggests an association [25,26]. ACEs may increase risk for excess adiposity via immune, metabolic, neuroendocrine, behavioral, and psychosocial responses to chronic or severe traumatic stress [5,12,13,14,15,16,17,18,19,20,21,22,23,24,25,26,27]. For example, ACEs-associated stress can lead to hypothalamic pituitary axis dysregulation and resulting cortisol attenuation that increases risk for weight gain [28]. ACEs effects can include insulin hypersecretion, insulin resistance, and hunger hormone dysregulation that leads to increased caloric intake and promote abdominal adiposity storage [28,29,30,31]. Mental health conditions resulting from ACEs exposure, including depression and binge eating disorder, also are risk factors for obesity [17,27,32,33]. ACEs-associated stress may lead to immune and metabolic derangements that promote excess adiposity, such as changes in the gut microbiome [34,35], as well as coping behaviors associated with obesity, such as overeating of highly palatable, calorically dense foods [27]. Additionally, household dysfunction associated with ACEs may detract from establishing routines and behaviors associated with maintaining body weight [27].

Most prior research on ACEs’ association with health outcomes, such as BMI, has focused at the individual- and family-levels. The role of the neighborhood environment has been understudied [36]. Consideration of neighborhood effects in ACEs research would align with multi-level theories of health, such as the socio-ecological model [37], as well as extensive empirical research that documents neighborhood effects (e.g., [38,39,40,41,42,43,44,45]). Multiple neighborhood characteristics, including neighborhood poverty, safety, greenspace, food access, alcohol outlet density, and physical and mental healthcare availability, are meaningfully and consistently associated with health [38,39,40,41,42,43,44,45] and could plausibly impact the association of ACEs with health outcomes. Consideration of the role of neighborhood provides context for how factors at higher levels of ecology may play a role in observed associations between ACEs and health outcomes. For example, neighborhood stressors such as poverty might exacerbate stress pathways underlying the ACEs-obesity association. Lack of access to greenspace might hinder nature-based stress reduction among populations exposed to ACEs, who often already experience a dysregulated stress response. Neighborhood crime might also inhibit outdoor physical activity. ACEs-associated cravings for highly palatable comfort foods may be amplified in a neighborhood with a plethora of fast-food restaurants. Lack of access to healthcare providers might limit ability to receive treatment for both ACEs sequelae, such as depression (an obesity risk factor), and obesity itself. Yet, none of these plausible neighborhood effects on the association of ACEs with health outcomes have been widely examined, highlighting a need for research focused on ACEs-neighborhood-health associations. Notably, a recent scoping review found that only 3 of 1175 ACEs studies focused on neighborhood conditions [36]. Overlooking the important role of neighborhood leaves an incomplete picture of how ACEs impact health, and hinders ability to inform upstream place-based interventions and policies to promote health for the millions of individuals affected by ACEs.

Illuminating neighborhood’s influence on ACEs’ association with health outcomes requires advanced spatial approaches that can account for the complexity of neighborhood environments. Neighborhoods are not simply comprised of risk factors in isolation. Neighborhoods are multi-faceted and synergistic environments encompassing many characteristics—safety, demographics, food environment, substance access, pollution, greenspace, and more [38,46,47]. Studies examining single neighborhood characteristics have value but cannot capture the collective influence of neighborhood on association between ACEs and health outcomes. Additionally, capturing neighborhood characteristics most associated with ACEs impact is challenging given that neighborhoods exist within a broader context of racial and economic segregation [48,49,50], resulting in co-occurrence and overlap of many potentially salient neighborhood characteristics (e.g., high crime, food deserts, low greenspace). Co-occurring neighborhood characteristics cannot be collectively examined using traditional methods such as regression, because inclusion of multiple correlated neighborhood characteristics as covariates in a regression model may result in problems associated with multicollinearity (e.g., model parameters with counterintuitive signs, elevated variance inflation factor). A composite metric that can incorporate numerous neighborhood characteristics simultaneously while accounting for and capturing their correlation, such as an index, is better suited to capture the collective impact of neighborhood environment. Neighborhood indexes are a single individual-level measure that can be applied to a dataset to capture neighborhood-level risk for each participant. An index approach has been employed in several widely used approaches for capturing neighborhood environments related to factors other than ACEs. For example, the “area deprivation index” is a composite measure of neighborhood socioeconomic conditions [51] that has been widely used to illuminate how neighborhood socioeconomics influence health outcomes such as cancer and heart failure [52,53]. 

Multiple methods for neighborhood index development exist and each could be applied to calculate the collective association of neighborhood characteristics with ACEs exposure. All index development methods begin with first collating and preparing a spatial dataset that reflects the multitude neighborhood characteristics (e.g., crime, alcohol outlet density, healthcare access) potentially associated with the outcome of interest (e.g., ACEs exposure), based on theory, prior research, and/or expert knowledge. Following creation of a spatial dataset, an index development method is applied to capture collective neighborhood environment. 

One analytic approach for developing a neighborhood index—and among the most widely used—is principal components analysis (PCA) [54,55,56,57]. PCA is a dimensionality reduction technique that attempts to capture the maximum information present in original data, while at the same time minimizing the error between the original data and the new lower dimensional representation. PCA is a linear method, meaning that the transformation between the two datasets is a linear projection. PCA output includes measures such as eigenvectors (linear representations of the spread of variance in a dataset), eigenvalues (standard deviations of each eigenvector), and loadings (measures of the magnitude of variance of each eigenvector explained by each variable) for each principal component (PC). The investigator specifies the number of components to use, often based on PCA output such as scree plots. The first component explains the most variation in the variables, with subsequent components describing amounts of variation in decreasing magnitude. 

PCA includes various sub-methods that can be employed to develop an index. PCA sub-methods all entail applying PCA to a dataset representing numerous exposure variables of interest (e.g., neighborhood characteristics). For example, a threshold-based method to index development entails consecutively repeating PCA on a dataset while applying a loading threshold to limit the number of variables in each subsequent analysis until further variable reduction is no longer possible. Another common method for using PCA for index development is to execute a PCA on the dataset and use the first PC as the index (rather than choosing the number of PCs based on a tool such a scree plots), because the first PC explains the most variation in the data and provides an easy to interpret single PC index. A third option is supervised PCA. Supervised PCA is a generalization of PCA which aims to determine the PCs with the greatest dependence on a response variable of interest. Supervised PCA is a unique method of PCA that differs from the aforementioned two PCA techniques, because it accounts for not only the exposure variables (e.g., neighborhood characteristics) but also the outcome of interest (e.g., ACEs exposure) [58].

In addition to PCA, a recently developed method that can be used to develop a neighborhood index is Bayesian index regression [59,60,61,62,63,64]. Bayesian index regression is an index development method grounded in weighted quantile sum regression and Bayesian methods. Since it entails a regression model, it offers possibility for covariate adjustment, comprehensive assessment of model performance, and incorporation of time varying and spatial random effects. Its quantile-based approach confers several additional benefits, including accounting for different scales of exposure variables, limiting the effect of outliers, decorrelating exposure variables, and acknowledging uncertainty in the exposure variables. Bayesian index regression takes into account both exposure variables (e.g., neighborhood characteristics) and an outcome variable (e.g., ACEs exposure), and estimates a weight for each exposure variable with weights being constrained to fall between 0 and 1 and sum to 1. Exposure variables with minimal association with the outcome receive weights near 0. Bayesian index regression may present several strengths as compared to PCA. First, its quantile-based approach and constraining of weights to 0–1 makes it well-suited to capture effects of multiple highly correlated neighborhood variables. Second, in contrast with most PCA methods, Bayesian index regression develops an index while incorporating information from both an exposure and the outcome of interest, as well as covariates and random effects as necessary. Third, Bayesian index regression results in easily interpretable weights, with weights for exposure variables that prove irrelevant being near 0; this is a contrast from PCA which may retain variables that are unrelated to the outcome of interest. Recent evidence suggests that Bayesian index regression performs better than traditional index development approaches such as PCA [59,64,65,66,67,68,69].

Neighborhood index development methods can be employed to create a “neighborhood ACEs index.” A neighborhood ACEs index is a composite measure that captures the association between neighborhood environment characteristics and individual-level ACEs exposure, within a particular population. Essentially, a neighborhood ACEs index answers the question “what is the neighborhood environment of individuals who have experienced ACEs?” Neighborhood ACEs index creation connects individual-level ACEs data with rich spatial data capturing numerous neighborhood characteristics such as crime, poverty, and healthcare access. A participant with a higher neighborhood ACEs index score lives in a neighborhood with more neighborhood risk factors associated with ACEs exposure. A neighborhood ACEs index can serve two purposes in ACEs research. First, it can help illuminate ACEs-neighborhood-health associations. More specifically, neighborhood ACEs index can be employed to answer the questions “what is the neighborhood environment of participants who have experienced ACEs?” and then “how does that neighborhood environment influence the ACEs-health associations observed in prior research?” For example, past studies document an association between ACEs and higher BMI, but it is plausible the association may change after accounting for neighborhood environment in which individuals with higher ACE scores live. Second, a neighborhood ACEs index can be used to identify population-specific neighborhood-level targets for interventions and polices, because index metrics quantify the extent to which each neighborhood characteristic is associated with ACEs within that sample, while accounting for collinearity among neighborhood characteristics. Research employing neighborhood ACEs indexes can build upon the strong, extensive ACEs evidence base that currently focuses primarily on the individual- and family-levels. 

The aim of this study was to evaluate methods for creation of a neighborhood ACEs index. This entailed comparing three PCA methods (threshold-based, first PC as index, and supervised PCA) and Bayesian index regression for the ability to create an index that explains ACEs exposure. The hypothesis was that Bayesian index regression would perform best. An exploratory aim was to examine the associations between ACEs, the neighborhood ACEs index, and BMI. The hypothesis was that the neighborhood ACEs index would be associated with higher BMI, both independently and after controlling for ACEs themselves. An additional hypothesis was that the neighborhood ACEs index would attenuate the association between BMI and ACEs. 

## 2. Materials and Methods

Study reporting is consistent with Strengthening the Reporting of Observational Studies in Epidemiology (STROBE) guidelines [70]. 

### 2.1. Study Design, Setting, and Sample

Study design entailed secondary analysis of existing cross-sectional survey and spatial data. Data included two sources: ACEs data and spatial neighborhood data. ACEs data were from the Philadelphia ACE Survey [2,11,71]. The Philadelphia ACE Survey was conducted as an add-on to the 2012 Southeastern Pennsylvania Household Health Survey. Randomly sampled Philadelphia residents 18 years of age and older were contacted by phone between November 2012 and January 2013 to participate in interviews on self-reported experience of 14 ACEs: physical, emotional, and sexual abuse; physical and emotional neglect; witnessing domestic violence; substance use, incarceration, or mental illness of a household member; witnessing community violence; racial/ethnic discrimination; low neighborhood safety; bullying; and foster care. The Philadelphia ACE Survey was informed by qualitative research with Philadelphia youth, expert task force input, prior literature, and existing tools for assessing ACE exposure. Additional detail about the Philadelphia ACE Survey can be found elsewhere [2,11,71]. In total, 1784 adults participated in the Philadelphia ACE Survey; this secondary analysis was focused on neighborhood environment and thus limited to participants with home census tract available in the data (*n* = 1679).

Spatial data reflecting Philadelphia neighborhood characteristics were collated from numerous publicly available sources, as detailed in Table 1. Data selection was guided by prior research, expert insight, and theory (the Healthy People 2020 Approach to Social Determinants of Health, a place-based framework for identifying the association between social determinants of health and a health outcome [e.g., ACEs]) [72]. Additionally, data selection was limited to variables likely publicly available in a standard measure for locations across the United States of America, in order to foster future replication of a neighborhood ACEs index in other samples and settings. When multiple years of spatial data were available, data were chosen that most closely reflected the date of the Philadelphia ACE Survey. 

### 2.2. Variables

Variables from the Philadelphia ACE Survey include ACEs exposure, BMI, participant characteristics, and home census tract. Number of ACEs experienced (potential range 0–14) was dichotomized as ≤3 versus 4+ ACEs, per evidence demonstrating 4+ ACEs is a meaningful threshold for conferring ACEs-related health risks [5,11]. BMI was calculated per self-reported height and weight and operationalized as a continuous variable. Participant characteristics and their operationalization included age (categorical: 18–34 [reference], 35–65, 65+), sex (dichotomous: male [reference], female), and race (categorical: Asian, Black, Hispanic, other, White [reference]). Participant’s home census tract at the time of survey participation was used as a proxy for neighborhood. The residential address was geocoded to census tract in the original Philadelphia ACE Survey data (i.e., prior to this secondary analysis).

Variables from the spatial data included 25 neighborhood variables, reflecting diverse aspects of neighborhood environment that may be important to consider when examining the role of in neighborhood in the association between ACEs and health. Included variables reflect neighborhood demographic makeup, resource availability, food environment, healthcare access, health status, alcohol access, crime, and outdoor environment. Variables were downloaded at and/or aggregated to the census tract level using ESRI ArcGIS 10.8 [74] and/or SAS 9.4 [75]. Table 1 details each neighborhood variable, its operationalization, and data source. A correlation matrix of neighborhood variables is presented in Supplementary Materials Table S1 in tabular format and Figure S1 via a visual representation.

### 2.3. Analyses: Comparison of Methods for Neighborhood ACEs Index Creation

Neighborhood spatial data were imported into RStudio 4.1.0 [76] and observations with missing data on any of the 25 neighborhood variables (*n* = 2 of 1679) were dropped from the dataset. Following, four neighborhood ACEs indexes were created, each using a potential index creation method (threshold-based PCA, first PC as index, supervised PCA, Bayesian index regression). The four neighborhood ACEs indexes were then compared using methods based on prior studies [59,64,65,66,67,68,69], described below. All index creation and comparison were executed using RStudio 4.1.0 [76]. 

### 2.4. Principal Components Analysis

Prior to each PCA method, variables were centered and scaled to ensure variables with large values did not dominate the PCA [54,55,56,57]. All variables were formatted to be in a direction consistent with higher values aligning with higher ACE exposure. Variables in the opposite direction were inverted using the formula max(x)-x_j_ where x_j_ was the value of the variable. Every PCA method used the spatial data that captured the neighborhood variables; the supervised PCA method also used the data that captured the binary ACE variable [54,55,56,57,58]. 

#### 2.4.1. Threshold-Based Principal Components Analysis

The first PCA method was the threshold-based method. The initial step entailed running a PCA limited to a two PC solution, using R function PRCOMP (within the STATS package). After the two PCA solution was executed, output was inspected including eigenvalues of each PC, proportion of variance explained by each PC, loadings of each variable on each PC, and number of variables with high loadings on each PC. (Of note, there is no widely accepted a priori threshold “high loading,” as loading magnitude is interpreted in a relative manner [54,55,56,57,58]. For this analysis, high loading was defined as a loading of ≥|0.15| with ≥|0.10| difference from loading on the other PCs.) Output demonstrated that each of the two PCs had at least four variables with high loading (a threshold chosen a priori to indicate meaningfulness of a PC). Thus following steps entailed repeating the PCA with increasing numbers of PCs (e.g., 3 PCs then 4 PCs etc.) until one of the resulting PCs had fewer than four variables with high loading. When that occurred, variables without high loading on any PC were dropped from the data and the process was repeated until no further variables merited dropping from that data. At that time, two PCs remained. Both PCs were considered collectively as one of the potential neighborhood ACEs indexes for comparison.

#### 2.4.2. First Principal Component as Index

The second PCA method entailed using the first PC as the index. A PCA was run using R function PRCOMP (within the STATS package). Output was inspected and the first PC was taken as one of the potential neighborhood ACEs indexes.

#### 2.4.3. Supervised Principal Components Analysis

The third PCA method was supervised PCA. A supervised PCA was run using R function SUPPCA (within the SUPERPCA package [77]). Output was inspected and the first PC was taken as one of the potential neighborhood ACEs indexes. 

### 2.5. Bayesian Index Regression

The fourth neighborhood ACEs index development approach was Bayesian index regression. The Bayesian index regression approach used the spatial data that captured the neighborhood variables and the data that captured the binary ACE variable. The Bayesian index regression model was estimated using a generalized linear model:

$$logit\left(p_{i}\right)=\beta_{0}+\beta_{1}\left(\overset{C}{\underset{j=1}{\sum }}w_{j},\;q_{ij}\right)$$

The neighborhood ACEs index reflected a weighted combination $\sum_{j}^{c}=1$ of quantiles $q_{i}\ldots q_{j}$ for the C neighborhood variables $x_{i}\ldots x_{C}$, with the weights $w_{i}\ldots w_{C}$ being estimated via the model. The number of quantiles was specified as 10 (e.g., deciles). The C = 25 neighborhood variables described in Table 1 were included. All variables were formatted to be in a direction consistent with higher values aligning with higher ACE exposure. The model was executed using R package BayesGWQS [78], which employs JAGS for analysis of Bayesian hierarchical models using Markov chain Monte Carlo (MCMC) algorithms. The package assigns a Dirichlet prior to variable weights, given that it constrains weights to be between 0 and 1 and ensures the weights sum to 1. The other model parameters are assigned vague normal priors: *β*_1_ *~*N($0,\;T_{1}$), precision $T_{1}$ *=* $1/\sigma_{1}^{2}$, and $\sigma_{1}\sim Uniform\;\left(0,100\right)$. Analysis included 10,000 MCMC iterations following 5000 burn-in, 500 adaptation interactions, a thinning parameter of 1, and 10 quantiles. Convergence was assessed using Geweke convergence criteria with absolute value of less than 2 considered evidence of convergence. Inference was conducted on the posterior medians of the model parameters. Statistical significance of the neighborhood ACEs index was assessed using the exponentiated *β* of its 95% credible interval, with the index considered significant if it did not contain 1.

### 2.6. Comparison of Methods for Neighborhood ACEs Index Creation

Our approach to comparing indexes aligned with approaches employed in prior studies [59,64,65,66,67,68,69]. To evaluate the best method for neighborhood ACEs index creation, each of the potential neighborhood ACEs indexes was applied to the data. Then, separate logistic regression models were run for each potential index with the dichotomous ACE score (≤3 versus 4+) as the dependent variable and the continuous neighborhood ACEs index as the independent variable. These models tested which index best explained experiencing 4+ ACEs. Models were compared based on Akaike Information Criteria (AIC), a goodness-of-fit measure that can be used in model selection. A lower AIC indicates a better model. In this study, the lowest AIC indicated the model whose neighborhood ACEs index best explained 4+ ACEs. The AIC is useful as a relative measure only, with a difference in AIC of ≥3 between models often considered meaningful [79]. Note that the Bayesian index model has an associated deviance information criterion [DIC] goodness-of-fit value also; a separate logistic regression model using the Bayesian index was carried out only to obtain an AIC value that would be comparable across all indexes.

### 2.7. Analyses: Association of Neighborhood ACEs Index with Health Outcome

The aim of this study was to evaluate methods for creation of a neighborhood ACEs index. An additional exploratory aim examined associations between ACEs, the neighborhood ACEs index, and a health outcome—in this case BMI. The exploratory aim answered two questions: (1) “is the neighborhood ACEs index [as a measure of the neighborhood environment of participants who have experienced ACEs] associated with BMI?” and (2) “does the association between ACEs and BMI change after accounting for the neighborhood ACEs index?”

To do so, first the neighborhood ACEs index chosen as the best index per the index comparison methods was applied to the data for each participant. Then, four linear multi-level regression models accounting for census tract-level clustering were run in RStudio 4.1.0 using package NLME. The first model examined the association of BMI (dependent variable) with the neighborhood ACEs index (independent variable); this model demonstrated whether the neighborhood ACEs index (as a composite measure of neighborhood environment associated with ACEs exposure) was associated with BMI. The second model examined the association of BMI (dependent variables) with the dichotomous ACEs variable (independent variable); this model demonstrated the association of ACEs with BMI without accounting for the neighborhood ACEs index. The third model examined the association of BMI (dependent variable) with the neighborhood ACEs index and dichotomous ACE variable (independent variables); this model demonstrated (1) whether an association of BMI with the neighborhood ACEs index persisted even after accounting for ACEs themselves and (2) whether the neighborhood ACEs index attenuated the association between BMI and ACEs. A fourth model examined the association of BMI (dependent variable) with the neighborhood ACEs index and ACEs (independent variables), after controlling for age, sex, and race; this model demonstrated whether the examined associations persisted after accounting for differences by age, sex, and race. For all models, ACEs, age, sex, race, BMI, and obesity were operationalized as described above under “variables;” the neighborhood ACEs index was a continuous variable. Of note, variance inflation factor was assessed for model 3 and model 4 to ensure inclusion of both ACEs and the neighborhood ACEs index did not violate collinearity assumptions. Statistical significance was assessed as *p* < 0.05.

Significance of neighborhood ACEs index in the models and a change in the ACEs-BMI association after controlling for the neighborhood ACEs index would suggest that neighborhood environment (as captured in the neighborhood ACEs index) may merit consideration in efforts to address the association of ACEs with that particular health outcome (in this case, BMI). Additionally, it would indicate a need for future research to more deeply examine aspects of the role of neighborhood such as timing of effects, subgroup differences, mediation and moderation effects, or influence of other individual, family, or neighborhood-level risk and protective factors.

## 3. Results

Overall, 1128 (67.3%) of participants experienced ≤3 ACEs and 547 (32.7%) experienced 4+ ACEs. The mean number of ACEs experienced was 3.8 ± 2.5, with a range of 0–13 (out of a possible range of 0–14). The majority of participants (*n* = 1216, 72.5%) reported being female. In this case, 183 (10.9%) were 18–34 years, 1016 (60.6%) were 35–64 years, and 476 (28.4%) were older than 65 years. Self-reported race/ethnicity was primarily White (786, 46.7%) and Black or African American (746, 44.5%), with 36 (2.1%) reporting Hispanic or Latino, 19 (1.1%) reporting Asian or Pacific Islander, and 54 (3.2%) reporting another race/ethnicity. Mean BMI was 29.3 ± 6.8 kg/m^2^, and *n* = 632 (37.7%) met criteria for obesity. 

### 3.1. Summary of Methods for Neighborhood ACEs Index Creation

Assessment of PCA output included examination of the resulting PC(s), including which variables were incorporated, variable loadings, and percent of variance explained. The first PCA method (threshold-based PCA) resulted in a neighborhood ACEs index that included 14 variables and two PCs (with eight having high loading on PC 1 and 6 having high loading on PC 2); PC 1 explained 37.8% of the variance and PC 2 explained 15.5% of the variance (total variance explained = 53.4%). The neighborhood variables with high loadings on PC 1 were violent crime, perceived poor mental health, poverty, martial support, perceived poor physical health, SNAP retailer access, greenspace, and mental healthcare diagnosis; the neighborhood variables with high loading on PC 2 were fast food access, transit access, mental healthcare access, substance use disorder treatment access, traffic burden, and alcohol access. As the final threshold-based solution included two PCs, each was considered to be part of that neighborhood ACEs index. The second PCA method (first PC as index) resulted in a neighborhood ACEs index with a PC that explained 36.4% of the variance and included all 25 variables. The third PCA method (supervised PCA) also resulted in a neighborhood ACEs index with one PC and included all 25 variables. For both, the five variables with the highest loadings were perceived poor mental health, poverty, violent crime, perceived poor physical health, and marital support. See Supplementary Materials Tables S2–S4 for details, including variable loadings for all variables for each method.

Assessment of the Bayesian index approach differed from the PCA methods, given that it was a regression-based approach to index development. Thus, assessment included examination of estimated variable weights, index *β* significance, and model convergence. The Bayesian index development approach resulted in a neighborhood ACEs index that included all 25 variables, with each variable weight ranging from 0.0189 to 0.1066. Ten of the 25 variable weights exceeded the weight that would have resulted from each of the 25 variables being applied an equal weight (0.04): supermarket access, unemployment, traffic burden, alcohol access, perceived poor mental health, poverty, marital support, perceived poor physical health, residential racial/ethnic segregation, and air quality. See Supplementary Materials Table S5 for details, including variable weights for all variables. The odds ratio for the neighborhood ACEs index’s association with the dichotomous ACE outcome was 1.24, suggesting 1.24 times greater odds of 4+ ACEs with each decile increase in the neighborhood ACEs index. The 95% credible interval of the odds ratio (1.14, 1.35) did not cross 1 which indicated statistical significance. Geweke convergence criteria indicated model convergence. 

### 3.2. Comparison of Methods for Neighborhood ACEs Index Creation

Model comparison results are presented in Table 2. The model with the best overall fit was the Bayesian index regression, with an AIC of 2107. The threshold-based PCA approach resulted in an index with two PCs. Thus, assessment of the threshold-based PCA approach entailed AIC from two models (one model for each PC). The PC #1 model had a fit that was slightly but not meaningfully higher (AIC of 2109), and the PC #2 model has a fit that was meaningfully higher (AIC of 2125) than the Bayesian index regression model. The model using the index from the first PC as index method resulted in an AIC of 2114 and the model using the index from supervised PCA method resulted in an AIC of 2114. Both were meaningfully higher than the Bayesian index regression model.

A difference in AIC of ≥3 is considered indicative of meaningfully better model fit [79]. Applying that threshold to these model comparisons demonstrates that the first PC as index method and the supervised PCA are do not have meaningfully better model fit than the best fitting approach (Bayesian index regression). The threshold-based PCA approach is comparable to the Bayesian index regression approach for its first PC only (AIC difference of +2), though not for its second PC (AIC difference of +18). Since assessment of the threshold-based PCA approach consisted of evaluation of both of its PCs, the threshold-based PCA approach was deemed collectively to have worse model fit than the Bayesian index regression approach.

### 3.3. Association of Neighborhood ACEs Index with Health Outcome

The neighborhood ACEs index from the Bayesian index regression was applied to the data, by multiplying posterior index weights for each neighborhood variable with that variable’s value for each participant. Models examining associations of the neighborhood ACEs index with BMI were then run and are presented in Table 3. Results demonstrated a significant association between BMI and the neighborhood ACEs index (model 1), and of BMI with ACEs (model 2). When the association between ACEs and the neighborhood ACEs index were considered collectively (model 3), the association between neighborhood ACEs index and BMI was slightly attenuated but remained significant; the association between ACEs and BMI was attenuated to a greater extent and no longer significant albeit close to the *p* < 0.05 threshold at *p* = 0.056 (model 3). Associations were similar after accounting for potential differences by age, race, and sex (model 4). Variance inflation factor demonstrating risk for collinearity between ACEs and the neighborhood ACEs index was within acceptable limits, being less than 2 for all models.

## 4. Discussion

This study sought to evaluate methods for creation of a neighborhood ACEs index. The recently developed method of Bayesian index regression led to the model with the best goodness-of-fit, which is consistent with results of other index methods comparison studies [59,64,65,66,67,68,69]. Exploratory analyses examining associations between ACEs, the neighborhood ACEs index, and BMI demonstrated that neighborhood environment associated with ACEs (as collectively captured in the neighborhood ACEs index) was associated with BMI, even after accounting for ACEs themselves. The neighborhood ACEs index attenuated the association between ACEs and BMI. Associations were consistent after accounting for potential differences by sex, race/ethnicity, and age. Future research can employ the methods established within this study to replicate and test a neighborhood ACEs index in other geographic settings and in application to other health outcomes. Such research can inform upstream, place-based interventions and policies to promote health among individuals affected by ACEs.

This work is grounded in the assertions that (1) neighborhoods are collective and complex environments containing unique features that can be useful in articulating risk for adverse health outcomes [38,46,47], and (2) that Bayesian index development is well-suited to operationalizing such neighborhood environments [59,60,61,62,63,64]. Neighborhoods are comprised of many complex and synergistic characteristics. Research focused on a single neighborhood characteristic (such as greenspace or supermarket access) has merit, but cannot capture collective aspects of the neighborhood environment nor account for collinearity among neighborhood characteristics that exist within a broader context of racial and economic segregation. Neighborhood environments are more than simply the sum of their parts. A complex, synergistic, and multi-faceted conceptualization of neighborhood has precedent in prior work in on neighborhood effects. For example, early research on “ecometrics” advocated for using more psychometrically sound methods with survey data to capture neighborhood characteristics related to factors such as socioeconomic position [80,81]; more recent work has applied ecometric approaches by using big data to measure neighborhood characteristics such as physical disorder [82]. Holistic approaches to measuring neighborhood, such as ecometrics, have advanced the field’s ability to consider neighborhood complexity and improve rigor of contextual measures. Individuals experience neighborhoods as collective environments and analytic approaches to index development can be harnessed to better reflect that reality. Such approaches must continue to be driven by empirical knowledge, theory, and expertise, which serves to guide selection of a comprehensive list of neighborhood characteristics included in index development. However, a benefit of the Bayesian index regression approach is that inclusion of hypothesis-driven neighborhood characteristics that may prove irrelevant is not a substantial risk, because irrelevant characteristics simply end up with a near zero weight [59,60,61,62,63,64]. As spatial data continue to increase in availability, opportunities for capturing collective measures of neighborhood environments using advanced analytic methods will continue to grow.

This study provides a tool for thinking about higher levels of ecology in ACEs research. Given the plethora of high-quality individual- and family-focused ACEs research, the field is now well-poised to build a body of evidence around how neighborhood characteristics and social determinants of health influence experiences of and outcomes associated with ACEs [36]. Consideration of neighborhood environment in ACEs-focused initiatives could align with other efforts to approach adversity at higher levels of ecology, such as creating trauma-informed built environments [83], trauma-informed community development [84,85], and focusing on how social determinants of health influence ACEs [36]. Doing so would align with efforts for promoting healing and well-being-promoting neighborhood spaces, such as therapeutic landscape theory [86,87] and could complement individual and family-level efforts to promote well-being among individuals who experience ACEs. All such efforts would require much additional research and thoughtful development. Stakeholder input would be key, including seeking the perspective of community members affected by ACEs about if/what neighborhood changes would be of interest to them. The neighborhood ACEs index provides one tool for beginning to build an evidence base that can inform this process. 

A neighborhood ACEs index could inform intervention and policy efforts to promote health among individuals affected by ACEs. For example, efforts to buffer ACEs’ affects could be targeted to neighborhoods where residents demonstrate the highest neighborhood ACEs index. Additionally, upstream policies and multi-level interventions to mitigate ACEs effects could be targeted to build environment factors identified as most salient per neighborhood ACEs index weights. A population whose neighborhood ACEs index identifies crime as the neighborhood characteristic with the greatest weight would likely require intervention tailoring that differs from that of a population whose index identifies low greenspace as having the greatest weight. Such interventions and policies would be most relevant in neighborhoods that bear a high ACEs burden, though efforts to focus on those neighborhoods would have to be carried out in a way that is sensitive to not perpetuating stigma associated with ACEs. Further, public service organizations working in areas relevant to ACEs, such as child welfare systems, could incorporate neighborhood ACEs index scores into their data to provide a single item summary measure of neighborhood-based risk for clients. 

A greater focus on the role of neighborhoods in ACEs research will inform efforts to take an equity-focused approach to addressing ACEs [36]. A better understanding of the neighborhood context associated with ACEs exposure can shift a narrative away from a narrow focus on an individual’s role in ACEs-associated outcomes and toward the potential influence of the environments in which individuals are experiencing ACEs-associated health risks. Structural factors that shape the neighborhood context could then be interrogated as an effort to promote upstream, trauma-informed policies that shift the inequities-promoting status quo [83]. An upstream approach recognizes ACEs not as a failing of parents, families, or individuals, but as occurring within a broader context of social determinants of health that synergize with and lead to disproportionate experience of ACEs [36]. Considering risk and protective factors at neighborhood-levels of ecology can inform a deeper understanding of the context in which ACEs occur and why inequities in both ACEs exposure and ACEs-associated outcomes are observed. Such understanding can signal additional evidence for the need to center equity when addressing ACEs.

More research is needed to illuminate how neighborhood influences the association between ACEs and health outcomes. For example, future research can test neighborhood ACEs indexes in longitudinal samples and cohorts reflecting populations who differ from adult Philadelphians. Studies can also examine how associations differ among subgroups, whether mediation or moderation effects exist, and the influence of individual, family, and neighborhood-level risk and protective factors. Additionally, this study focused on BMI as the outcome of interest. Given that ACEs are associated with numerous health behaviors and conditions (e.g., smoking, alcohol use, depression cancer, cardiovascular disease) [5,6,8] other than obesity, future work examining neighborhood ACEs indexes with other physical and mental health outcomes would be informative. In addition, qualitative work with individuals affected by ACEs about their perspectives on how neighborhoods influence their health could provide context surrounding a neighborhood ACEs index. Additionally, a focused review of neighborhood characteristics associated with highest index weights can inform most salient neighborhood-level targets for place-based intervention and policy development. Lastly, while this study evaluated methods for neighborhood ACEs index creation using Philadelphia data, the authors invite replication across datasets with diverse samples, geographies, and areas of focus related to ACEs. 

Our study has limitations. Analyses employed secondary data; results may have differed using other sources. The analytic decisions, such as selection of neighborhood variables, and choice of analytic methods to compare, were ground in prior research, theory, and the authorship team’s content expertise. However, findings may have differed if other decisions were made or if other analytic approaches were tested. For example, use of a dimensionality reduction technique other than PCA may have changed study results. Additionally, limitation of our neighborhood variables to those that were publicly available for locations across the United States of America meant that measures that are important to neighborhood context but not available as a comprehensive or standard national measure, such as collective efficacy or social cohesion, could not be included. Our choice of ACEs threshold (≤3 versus 4+) aligned with norms in the field and was ground in prior research; however, it is possible a different threshold may have resulted in different results, particularly given that the Philadelphia ACE Survey includes a more comprehensive list of ACEs than much prior research on which the threshold is based. Findings may not be generalizable outside Philadelphia, though the authors invite replication of the neighborhood ACEs index in different settings. Illustrative analyses of associations with BMI were cross-sectional and cannot support causal inference. Lastly, census tract was employed as a proxy for neighborhood. However use of census tracts as a measure of neighborhood has several limitations, including that (1) census tracts are administrative boundaries that may not reflect the lived realities of where participants perceive to be their neighborhoods, (2) it ignores variation over space within census tracts, (3) boundary issues may exist based upon census tracts’ arbitrary borders, (4) it is susceptible to the modifiable areal unit problem in which small areas are sensitive to scale and aggregation, and (5) census tract of home residence may not accurately reflect the spaces in which individuals actually spend much of their time [79,88,89,90].

## 5. Conclusions

Most research on ACEs has focused at the individual and family-levels, presenting a need and opportunity to explore ACEs-neighborhood-health associations. This study tested methods for creation of a neighborhood ACEs index, a tool that can be applied in future research to illuminate the association between ACEs, neighborhood, environment, and health outcomes. Future research can employ neighborhood ACEs index to inform upstream place-based interventions and polices to promote health among individuals affected by ACEs.

## Figures and Tables

**Table 1:** Operationalization and data source for neighborhood variables included in neighborhood ACEs index.

Neighborhood Variable	Operational Definition	Data Source
Neighborhood Demographic Makeup and Socioeconomic Resource Access		
Residential racial/ethnic segregation	% of population who identify as African American, Hispanic/Latino, Asian, multiracial, or any race other than White. Higher value indicates higher % who identify as race other than White in that census tract. Potential range 0–100.	United States Census American Community Survey (ACS) ^a^
Language Proficiency	% of population ≥5 years speaking English less than very well. Higher value indicates higher % speaking English less than very well in that census tract. Potential range 0–100.	United States Census ACS ^a^
Unemployment	% of population age ≥16 years in labor force who were unemployed. Higher value indicates higher % unemployed in that census tract. Potential range 0–100.	United States Census ACS ^a^
Education	% of population with less than a high school education. Higher value indicates higher % with less than high school education in that census tract. Potential range 0–100.	United States Census ACS ^a^
Poverty	% of population below federal poverty level. Higher value indicates higher % of population below federal poverty level in that census tract. Potential range 0–100.	United States Census ACS ^a^
Homeownership ^b^	% of households that are owner-occupied. Higher value indicates higher % owner-occupied in that census tract. Potential range 0–100.	United States Census ACS ^a^
Internet access ^b^	% of households with internet access. Higher value indicates higher % with internet access in that census tract. Potential range 0–100.	United States Census ACS ^a^
Marital support ^b^	% of people older than 15 years who are married. Higher value indicates higher % married in that census tract. Potential range 0–100.	United States Census ACS ^a^
**Neighborhood Healthy and Unhealthy Food Availability**		
Fast-Food Access ^b^	# fast-food restaurants with >$0 in sales per 1000 people. Higher value indicates higher # of fast-food restaurants in that census tract. Potential range ≥ 0.	National Neighborhood Data Archive—University of Michigan, Inter-university Consortium for Political and Social Research
SNAP Retailer Access	# stores authorized to accept the Supplemental Nutrition Assistance Program (SNAP) per 10,000 residents. Higher value indicates higher # of SNAP stores in that census tract. Potential range ≥ 0.	United States Department of Agriculture Food and Nutrition Service ^a^
Supermarket Access	Low supermarket access score: % by which that tract’s distance to the nearest supermarket would have to be reduced to equal the typical distance for well-served census tract. Higher value indicates higher % reduction required (e.g., higher value indicates worse supermarket access) in that census tract. Potential range 0–100.	Reinvestment Fund ^a^
**Neighborhood Healthcare Access**		
Health Insurance	% of population without health insurance. Higher value indicates higher % without health insurance in that census tract. Potential range 0–100.	United States Census ACS ^a^
Healthcare Access for Uninsured	# federally qualified and community health centers per 10,000 people. Higher value indicates higher # of centers per 10,000 people in that census tract. Potential range ≥ 0.	Health Resources and Services Administration ^a^
Mental Healthcare Access	# mental healthcare facilities per 10,000 people. Higher value indicates higher # of facilities per 10,000 people in that census tract. Potential range ≥ 0.	Substance Abuse and Mental Health Services Administration (SAMHSA) ^a^
Substance Use Disorder Treatment Access	# substance use disorder treatment facilities per 10,000 people. Higher value indicates higher # of facilities per 10,000 people in that census tract. Potential range ≥ 0.	SAMHSA ^a^
Mental Healthcare Diagnosis ^b^	% of adults ever diagnosed with depression. Higher value indicates higher % ever diagnosed with depression in that census tract. Potential range 0–100.	CDC Behavioral Risk Factor Surveillance System (BRFSS); United States Census Survey ACS ^a^
**Neighborhood Health Status**		
Perceived Poor Mental Health	% of adults reporting ≥ 7 days of poor mental health in past 30 days. Higher value indicates higher % reporting poor mental health in that census tract. Potential range 0–100.	CDC BRFSS; United States Census ACS ^a^
Perceived Poor Physical Health	% of adults reporting ≥ 7 days of poor physical health in past 30 days. Higher value indicates higher % reporting poor physical health in that census tract. Potential range 0–100.	CDC BRFSS; United States Census ACS ^a^
**Neighborhood Alcohol Access**		
Alcohol Access	# alcohol outlets for to-go purchase per 10,000 people. Higher value indicates higher # of outlets per 10,000 people in that census tract. Potential range ≥ 0.	State Liquor Control Board
**Neighborhood Crime**		
Non-violent Crime	# non-violent crimes (e.g., prostitution, gambling, fraud) reported per 10,000 people Higher value indicates higher # of non-violent crimes per 10,000 people in that census tract. Potential range ≥ 0.	Police department
Violent Crime	# violent crimes (e.g., aggravated assault, rape, arson) reported per 10,000 people. Higher value indicates higher # of violent crimes per 10,000 people in that census tract. Potential range ≥ 0.	Police department
**Neighborhood Transit Environment**		
Traffic Burden ^b^	%tile of count of vehicles at major roads per meter within 500 m, as compared to USA. Higher value indicates higher %tile (e.g., higher value indicates more traffic) in that census tract. Potential range 0–100.	Environmental Protection Agency EJSCREEN Environmental Justice Screening and Mapping Tool
Transit Access ^b^	Frequency of transit service per hour within 0.25 miles Higher value indicates higher frequency of transit services in that census tract. Potential range ≥ 0.	Environmental Protection Agency ^a^
**Neighborhood Outdoor Quality**		
Greenspace ^b^	% of land that is urban greenspace. Higher value indicates higher % greenspace in that census tract. Potential range 0–100.	US Geological Survey National Land Cover Database
Air Quality	%tile PM2.5 levels (µg/m^3^ annual average) versus national average. Higher value indicates higher %tile (e.g., higher value indicates worse air quality) in that census tract. Potential range 0–100.	Environmental Protection Agency EJSCREEN Environmental Justice Screening and Mapping Tool

Note: “%” indicates “percent” and “#” indicates “number. ^a^ Data sourced via PolicyMap spatial data and analytics platform [73] ^b^ For neighborhood ACEs index creation, all neighborhood variables were formatted to be in a direction consistent with higher values aligning with higher ACE exposure. Variables in the opposite direction were inverted using the formula max(x)-x_j_ where x_j_ was the value of the variable. Such variables are noted with “^b^” in the table.

**Table 2:** Comparison of methods for development of neighborhood ACEs index.

Method for Neighborhood ACEs Index Development	AIC (Lower Is Better)
Principal components analysis: Threshold-based PC #1	2109
Principal components analysis: Threshold-based PC #2	2125
Principal components analysis: First PC as index	2114
Supervised principal components analysis	2114
Bayesian index regression	2107

Note: AIC = Akaike information criteria. PC = Principal component. Model outcome was the binary ACE variable (≤3 versus 4+). Analytic approach was logistic regression.

**Table 3:** Results of models examining associations of neighborhood ACEs index and ACEs with BMI.

Model	β (95% CI)	*p*-Value
Model 1		
Neighborhood ACEs Index	0.037 (0.024, 0.050)	<0.001
**Model 2**		
4+ ACEs (Reference: Yes)	0.847 (0.142, 1.551)	0.0185
**Model 3**		
Neighborhood ACEs Index 4+ ACEs (Reference: Yes)	0.036 (0.023, 0.049) 0.684 (−0.018, 1.386)	<0.001 0.056
**Model 4**		
Neighborhood ACEs index	0.021 (0.007, 0.035)	0.003
4+ ACEs (Reference: Yes)	0.427 (−0.289, 1.143)	0.242
Male (Reference: Female)	0.518 (−0.216, 1.251)	0.167
Race/ethnicity (Reference: White)		
Black or African American	2.210 (1.458, 2.961)	<0.001
Hispanic or Latino	−0.228 (−2.550, 2.094)	0.847
Asian or Pacific Islander	−3.047 (−6.107, 0.012)	0.051
Other	1.508 (−0.372, 3.388)	0.116
Age (Reference: 18–34)		
35–64	1.795 (0.703, 2.886)	0.001
65+	0.481 (−0.709, 1.671)	0.428

Note: ACE = adverse childhood experiences. BMI = body mass index. Analytic approach was multi-level linear regression models accounting for clustering at census tract-level.

## Data Availability

Philadelphia ACE Project data is not publicly available. All spatial data used in the study are publicly available; see manuscript Table 1 for details.

## Supplementary Materials

The following supporting information can be downloaded at: https://www.mdpi.com/article/10.3390/ijerph19137819/s1, Table S1: Correlation matrix of neighborhood variables; Table S2: Neighborhood variable loadings for threshold-based principal components analysis approach; Table S3: Neighborhood variable loadings for first principal component as index principal components analysis approach; Table S4: Neighborhood variable loadings for supervised principal components analysis approach; Table S5: Neighborhood variable weights for Bayesian index regression approach; Figure S1: Visualization of correlation matrix of neighborhood variables.
