# Supplementary material for: The Intersection of Neighborhood Environment and Adverse Childhood Experiences: Methods for Creation of a Neighborhood ACEs Index
Source: Int J Environ Res Public Health. 2022 Jun 25;19(13):7819. doi: 10.3390/ijerph19137819 (PMC9265402; doi:10.3390/ijerph19137819)
Supplement: Supplementary file 1 [file ijerph-19-07819-s001.zip › ijerph-1726216-supplementary.pdf]

Supplementary File S1

“The intersection of neighborhood environment and adverse childhood experiences: Methods for creation of a neighborhood ACEs index”

Table S1. Correlation matrix of neighborhood variables

	alcohol access	supermarket access	greenspace	substance use treatment access	poverty	unemployment	health insurance	education	internet access	mental healthcare access	air quality	perceived poor mental health	perceived poor physical health	nonviolent crime	marital support	language proficiency	racial ethnic segregation	violent crime	healthcare access for uninsured	fast-food access	snap retailer access	mental healthcare diagnosis	homeownership	transit access	traffic burden
**alcohol access**	1.00	-0.08	-0.14	0.08	0.04	-0.03	-0.08	-0.03	-0.03	0.07	0.16	0.10	0.03	0.25	-0.09	-0.03	-0.05	0.17	0.14	0.25	0.30	0.01	-0.21	0.23	0.11
**supermarket access**	-0.08	1.00	-0.34	0.09	0.37	0.34	0.29	0.37	-0.35	0.08	0.30	0.39	0.40	0.18	-0.40	0.03	0.33	0.32	0.07	-0.08	0.23	-0.25	-0.21	0.03	0.17
**greenspace**	-0.14	-0.34	1.00	-0.08	-0.47	-0.37	-0.34	-0.44	0.36	-0.09	-0.63	-0.49	-0.35	-0.39	0.51	-0.17	-0.28	-0.46	-0.20	-0.15	-0.46	0.22	0.27	-0.22	-0.28
**substance use disorder treatment access**	0.08	0.09	-0.08	1.00	0.10	0.09	-0.07	0.00	-0.09	0.64	0.16	0.12	0.15	0.21	-0.11	-0.03	0.04	0.25	0.29	0.24	0.15	-0.12	-0.24	0.20	0.08
**poverty**	0.04	0.37	-0.47	0.10	1.00	0.69	0.43	0.71	-0.65	0.11	0.48	0.92	0.76	0.53	-0.72	0.16	0.61	0.71	0.26	-0.05	0.51	-0.41	-0.51	0.14	0.01
**unemployment**	-0.03	0.34	-0.37	0.09	0.69	1.00	0.49	0.61	-0.66	0.10	0.26	0.65	0.70	0.39	-0.56	0.05	0.60	0.59	0.14	-0.11	0.48	-0.40	-0.17	-0.07	-0.05
**health insurance**	-0.08	0.29	-0.34	-0.07	0.43	0.49	1.00	0.58	-0.46	-0.01	0.21	0.46	0.40	0.18	-0.31	0.38	0.48	0.32	0.01	-0.17	0.38	-0.36	-0.14	-0.15	-0.05
**education**	-0.03	0.37	-0.44	0.00	0.71	0.61	0.58	1.00	-0.60	0.04	0.29	0.66	0.63	0.35	-0.46	0.49	0.46	0.53	0.13	-0.16	0.49	-0.35	-0.22	-0.04	-0.01
**internet access**	-0.03	-0.35	0.36	-0.09	-0.65	-0.66	-0.46	-0.60	1.00	-0.12	-0.27	-0.61	-0.81	-0.41	0.59	-0.02	-0.70	-0.64	-0.18	0.13	-0.50	0.55	0.26	0.02	0.06
**mental healthcare access**	0.07	0.08	-0.09	0.64	0.11	0.10	-0.01	0.04	-0.12	1.00	0.14	0.12	0.11	0.23	-0.09	0.02	0.03	0.27	0.28	0.22	0.18	-0.07	-0.21	0.18	0.11
**air quality**	0.16	0.30	-0.63	0.16	0.48	0.26	0.21	0.29	-0.27	0.14	1.00	0.52	0.34	0.45	-0.60	-0.02	0.38	0.49	0.26	0.23	0.39	-0.34	-0.52	0.44	0.19
**perceived poor mental health**	0.10	0.39	-0.49	0.12	0.92	0.65	0.46	0.66	-0.61	0.12	0.52	1.00	0.76	0.55	-0.74	0.11	0.59	0.71	0.27	-0.01	0.52	-0.37	-0.61	0.16	0.06
**perceived poor physical health**	0.03	0.40	-0.35	0.15	0.76	0.70	0.40	0.63	-0.81	0.11	0.34	0.76	1.00	0.48	-0.64	0.00	0.70	0.67	0.23	-0.01	0.54	-0.60	-0.34	0.06	-0.03
**nonviolent crime**	0.25	0.18	-0.39	0.21	0.53	0.39	0.18	0.35	-0.41	0.23	0.45	0.55	0.48	1.00	-0.55	-0.05	0.32	0.85	0.42	0.48	0.67	-0.30	-0.37	0.61	0.09
**marital support**	-0.09	-0.40	0.51	-0.11	-0.72	-0.56	-0.31	-0.46	0.59	-0.09	-0.60	-0.74	-0.64	-0.55	1.00	0.23	-0.68	-0.71	-0.26	-0.05	-0.43	0.44	0.49	-0.28	-0.06
**language proficiency**	-0.03	0.03	-0.17	-0.03	0.16	0.05	0.38	0.49	-0.02	0.02	-0.02	0.11	0.00	-0.05	0.23	1.00	-0.10	-0.04	-0.02	-0.03	0.17	-0.11	-0.06	-0.06	0.04
**racial ethnic segregation**	-0.05	0.33	-0.28	0.04	0.61	0.60	0.48	0.46	-0.70	0.03	0.38	0.59	0.70	0.32	-0.68	-0.10	1.00	0.57	0.15	-0.18	0.39	-0.80	-0.25	0.02	-0.20
**violent crime**	0.17	0.32	-0.46	0.25	0.71	0.59	0.32	0.53	-0.64	0.27	0.49	0.71	0.67	0.85	-0.71	-0.04	0.57	1.00	0.39	0.23	0.70	-0.46	-0.42	0.42	0.07
**healthcare access for uninsured**	0.14	0.07	-0.20	0.29	0.26	0.14	0.01	0.13	-0.18	0.28	0.26	0.27	0.23	0.42	-0.26	-0.02	0.15	0.39	1.00	0.33	0.30	-0.17	-0.29	0.29	0.06
**Fast-food access**	0.25	-0.08	-0.15	0.24	-0.05	-0.11	-0.17	-0.16	0.13	0.22	0.23	-0.01	-0.01	0.48	-0.05	-0.03	-0.18	0.23	0.33	1.00	0.37	-0.07	-0.15	0.60	0.24
**snap retailer access**	0.30	0.23	-0.46	0.15	0.51	0.48	0.38	0.49	-0.50	0.18	0.39	0.52	0.54	0.67	-0.43	0.17	0.39	0.70	0.30	0.37	1.00	-0.40	-0.32	0.29	0.07
**mental healthcare diagnosis**	0.01	-0.25	0.22	-0.12	-0.41	-0.40	-0.36	-0.35	0.55	-0.07	-0.34	-0.37	-0.60	-0.30	0.44	-0.11	-0.80	-0.46	-0.17	-0.07	-0.40	1.00	0.27	-0.24	0.13
**homeownership**	-0.21	-0.21	0.27	-0.24	-0.51	-0.17	-0.14	-0.22	0.26	-0.21	-0.52	-0.61	-0.34	-0.37	0.49	-0.06	-0.25	-0.42	-0.29	-0.15	-0.32	0.27	1.00	-0.37	-0.18
**transit access**	0.23	0.03	-0.22	0.20	0.14	-0.07	-0.15	-0.04	0.02	0.18	0.44	0.16	0.06	0.61	-0.28	-0.06	0.02	0.42	0.29	0.60	0.29	-0.24	-0.37	1.00	0.28
**traffic burden**	0.11	0.17	-0.28	0.08	0.01	-0.05	-0.05	-0.01	0.06	0.11	0.19	0.06	-0.03	0.09	-0.06	0.04	-0.20	0.07	0.06	0.24	0.07	0.13	-0.18	0.28	1.00

Note: See manuscript for additional detail, including neighborhood variable descriptions.

Figure S1: Visualization of correlation matrix of neighborhood variables

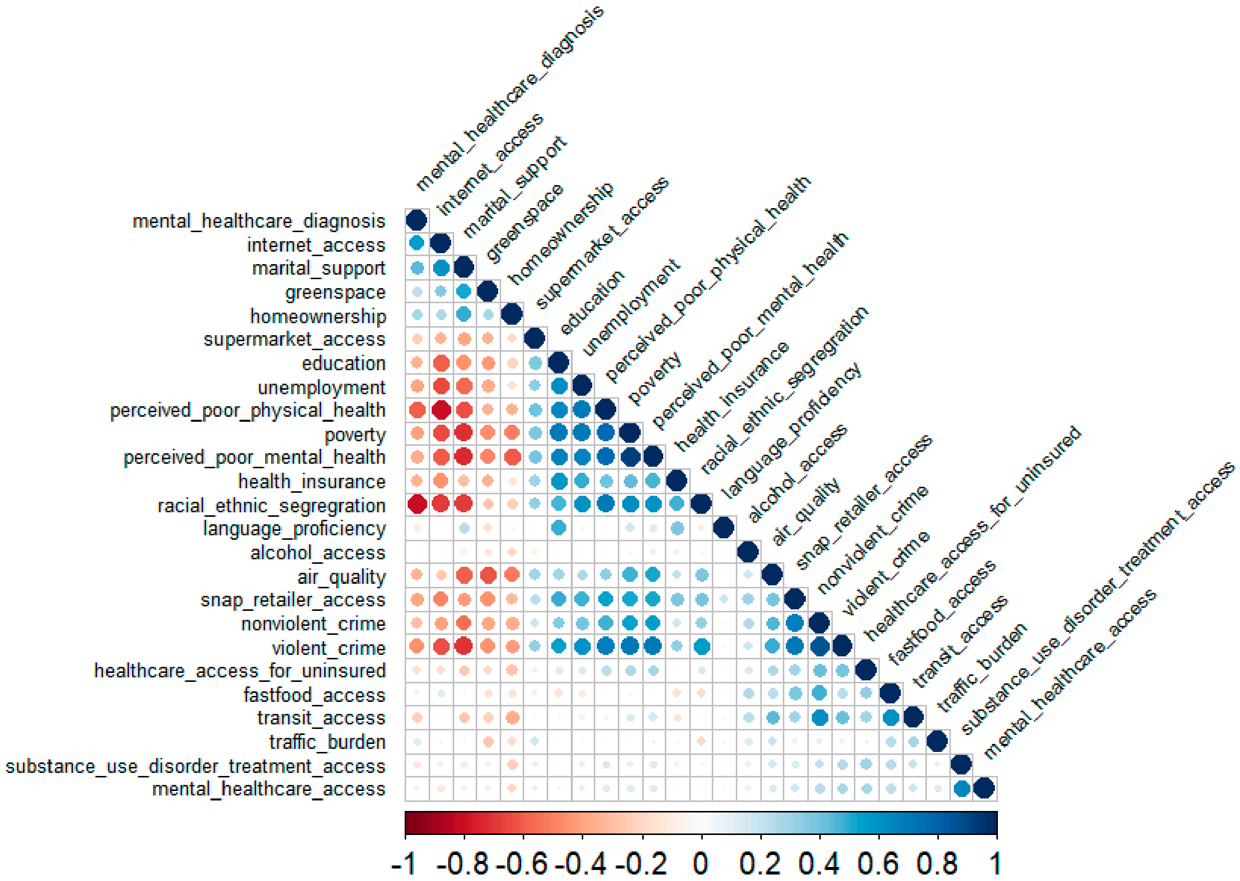

Table S2. Neighborhood variable loadings for threshold-based principal components analysis approach

Neighborhood Variable	Loading	
	Principal Component 1	Principal Component 2
Violent crime	-0.38745	-0.03697
Perceived poor mental health	-0.37428	0.19064
Poverty	-0.36989	0.22825
Marital support (inverted)	-0.35480	0.12664
Perceived poor physical health	-0.35312	0.22492
SNAP retailer access	-0.32184	-0.10951
Greenspace (inverted)	-0.26771	-0.03740
Mental healthcare diagnosis (inverted)	-0.25494	0.10883
Transit access (inverted)	-0.17676	-0.41947
Substance use disorder treatment access	-0.12115	-0.33278
Mental healthcare access	-0.11746	-0.33488
Alcohol access	-0.08954	-0.26212
Traffic burden (inverted)	0.05609	0.30142
Fast-food access (inverted)	0.10617	0.50984

Note: See manuscript for additional detail, including neighborhood variable descriptions. For neighborhood ACEs index creation, all neighborhood variables were formatted to be in a direction consistent with higher values aligning with higher ACE exposure. Variables in the opposite direction were inverted using the formula max(x)-x_j where x_j was the value of the variable. Such variables are noted as “(inverted)” in the table.

Table S3. Neighborhood variable loadings for first principal component as index principal components analysis approach

Neighborhood Variable	Loading
Perceived poor mental health	0.2899863112
Poverty	0.2882607837
Violent crime	0.2881907051
Perceived poor physical health	0.2775788057
Marital support (inverted)	0.2701629765
Internet access (inverted)	0.2553740887
Residential racial/ethnic segregation	0.2432133801
Unemployment	0.2419010488
SNAP retailer access	0.2362162949
Education	0.2342372146
Nonviolent crime	0.2335219157
Air quality	0.2055577676
Mental healthcare diagnosis (inverted)	0.2040244034
Greenspace (inverted)	0.1999842342
Homeownership (inverted)	0.1799530828
Health insurance	0.1727465487
Supermarket access	0.1549618760
Healthcare access for uninsured	0.1230165886
Transit access (inverted)	0.1016744410
Mental healthcare access	0.0728338566
Substance use disorder treatment access	0.0719526596
Alcohol access	0.0453220389
Language proficiency	0.0330910609
Traffic burden (inverted)	-0.0236871193
Fast-food access (inverted)	-0.0431860790

Note: See manuscript for additional detail, including neighborhood variable descriptions. For neighborhood ACEs index creation, all neighborhood variables were formatted to be in a direction consistent with higher values aligning with higher ACE exposure. Variables in the opposite direction were inverted using the formula max(x)-x_j where x_j was the value of the variable. Such variables are noted as "(inverted)" in the table.

Table S4. Neighborhood variable loadings for supervised principal components analysis approach

Neighborhood Variable	Loadings
Perceived poor mental health	0.2900161750
Poverty	0.2883038296
Violent crime	0.2881024032
Perceived poor physical health	0.2775794591
Marital support (inverted)	0.2702026654
Internet access (inverted)	0.2554073466
Residential racial/ethnic segregation	0.2433167642
Unemployment	0.2420350126
SNAP retailer access	0.2360890465
Education	0.2343450441
Nonviolent crime	0.2333150829
Air quality	0.2055647773
Mental healthcare diagnosis (inverted)	0.2040047877
Greenspace (inverted)	0.1999493451
Homeownership (inverted)	0.1798855900
Health insurance	0.1728040615
Supermarket access	0.1551874591
Healthcare access for uninsured	0.1228727244
Transit access (inverted)	0.1015260849
Mental healthcare access	0.0727880838
Substance use disorder treatment access	0.0719485599
Alcohol access	0.0454388895
Language proficiency	0.0331041207
Traffic burden (inverted)	-0.0235188241
Fast-food access (inverted)	-0.0429972571

Note: See manuscript for additional detail, including neighborhood variable descriptions. For neighborhood ACEs index creation, all neighborhood variables were formatted to be in a direction consistent with higher values aligning with higher ACE exposure. Variables in the opposite direction were inverted using the formula max(x)-x_j where x_j was the value of the variable. Such variables are noted as "(inverted)" in the table.

Table S5. Neighborhood variable weights for Bayesian index regression approach

Neighborhood Variable	Weight
Supermarket access	0.1065623321
Unemployment	0.0914996541
Traffic burden (inverted)	0.0623819095
Alcohol access	0.0559543316
Perceived poor mental health	0.0522796754
Poverty	0.0506823784
Marital support (inverted)	0.0481190075
Perceived poor physical health	0.0431961483
Residential racial/ethnic segregation	0.0404674960
Air quality	0.0404489053
Internet access (inverted)	0.0396487989
Education	0.0356445498
Violent crime	0.0336969638
Homeownership (inverted)	0.0301164498
Mental healthcare diagnosis (inverted)	0.0297516256
Language proficiency	0.0282183699
Greenspace (inverted)	0.0279695854
Health insurance	0.0275390117
Nonviolent crime	0.0267914376
Transit access (inverted)	0.0266842109
Fast-food access (inverted)	0.0241971341
SNAP retailer access	0.0211281396
Substance use disorder treatment access	0.0190856903
Mental healthcare access	0.0190099901
Healthcare access for uninsured	0.0189262041

Note: See manuscript for additional detail, including neighborhood variable descriptions. For neighborhood ACEs index creation, all neighborhood variables were formatted to be in a direction consistent with higher values aligning with higher ACE exposure. Variables in the opposite direction were inverted using the formula max(x)-x_j where x_j was the value of the variable. Such variables are noted as "(inverted)" in the table.
